# Pseudotumor Tensor Fascia Lata Syndrome From Underlying Severe Lumbar Stenosis: A Case Report

**DOI:** 10.7759/cureus.80523

**Published:** 2025-03-13

**Authors:** Zane Shah, Won J Jeong, Wyatt I Kupperman, Yonghoon Lee

**Affiliations:** 1 Physical Medicine and Rehabilitation, Baylor College of Medicine, Houston, USA

**Keywords:** botulinum toxin therapy, denervation hypertrophy, diagnostic mri, hip abductors, lumbar spinal stenosis, mechanical hypertrophy, soft tissue mass, tensor fascia lata hypertrophy, tensor fascia lata pseudohypertrophy, tensor fascia lata pseudotumor

## Abstract

A pseudotumor of the tensor fascia lata (TFL) describes a rare condition characterized by hypertrophy or pseudohypertrophy of the TFL that can mimic a soft tissue mass. Without the right clinical suspicion, it can be misdiagnosed as a benign or malignant soft tissue tumor, leading to oncologic workups. Unnecessary workups can cause patient distress, waste resources, and delay the management of the condition and its associated symptoms. Here, we describe a 65-year-old male who developed a pseudotumor of the TFL in the setting of severe lumbar spinal stenosis. The etiology for this patient was suspected to be a combination of altered gait mechanics and denervation secondary to L4/5 radiculopathy. There is a paucity of current research, literature, and clinical cases on this topic. However, the prior cases share similarities in presentation and imaging findings. Magnetic resonance imaging (MRI) is the gold standard for diagnostic confirmation in nearly all cases, with treatment ranging from hip abductor strengthening to botulinum toxin injections. To limit unnecessary diagnostic testing and invasive procedures, it is important for clinicians to be aware of TFL hypertrophy and its possible etiologies.

## Introduction

Pseudotumor of the tensor fascia lata (TFL) describes a rare condition characterized by hypertrophy of the TFL that can mimic a soft tissue mass. The etiology is not entirely understood, but two theories are suggested in the literature: mechanical hypertrophy, where altered biomechanics lead to compensatory hypertrophy of the TFL, and denervation hypertrophy, where a muscle unexpectedly enlarges following the loss of innervation [1,2]. Mechanical hypertrophy can result from the overuse of a specific muscle or group of muscles as a result of changes in a patient's gait, while the exact mechanism of denervation hypertrophy is not well understood. On magnetic resonance imaging (MRI), true muscle hypertrophy appears as an isointense enlargement of normal-appearing muscle fibers, while pseudohypertrophy appears as an enlargement of muscle with excessive amounts of fat interspersed throughout the muscle [2]. Without proper clinical suspicion, it can be misdiagnosed as a benign or malignant soft tissue tumor, leading to unnecessary oncologic workups that can cause patient distress and waste resources. Here, we describe a 65-year-old male who developed a pseudotumor of the TFL in the setting of lumbar spinal stenosis. This case adds to the existing knowledge regarding this rare condition, including its etiology, evaluation, and management for appropriate patient care.

## Case presentation

A 65-year-old male with a history of diffuse idiopathic skeletal hyperostosis (DISH) and chronic low back pain for many years presented to a Physical Medicine and Rehabilitation (PM&R) clinic with acute-on-chronic right gluteal and sacroiliac joint (SIJ) pain, which had been constant for six months with occasional episodes of worsening pain. The pain was rated as five out of 10 on a Visual Analog Scale. The patient reported that the pain improved with mild physical activity and analgesic medications, such as gabapentin and acetaminophen, but worsened with strenuous or prolonged exertion. He denied any symptoms of neurogenic claudication, motor weakness, bowel and bladder dysfunctions, or saddle anesthesia. 

On physical exam, tenderness was noted in the right mid to distal SIJ region, along with decreased lumbar spine and hip range of motion. Strength and sensation in the upper and lower extremities were intact. Various maneuvers, namely Faber, Yeoman’s, and sacral compression tests, were positive. MRI of the lumbar spine showed moderately severe central stenosis at L4-L5 as well as mild and moderately severe bilateral foraminal narrowing at L3-L4 and L4-L5, respectively. The patient was diagnosed with chronic low back pain, likely due to SIJ-referred pain and/or L4/5 radiculopathy, and was treated with physical therapy, a home exercise program, and right L4-L5 transforaminal epidural steroid injections. At follow-up visits, the patient reported symptom improvement with this comprehensive treatment plan. 

However, 15 months after the initial consultation, the patient reported new symptoms, including swelling and discomfort in the right lateral gluteal region. Examination revealed a notable palm-sized fullness at the anterior and lateral right greater trochanter region with mild discomfort on palpation. Internal and external rotation of the hip was decreased. Diagnostic ultrasound performed in the clinic revealed a large, well-demarcated soft tissue mass or hypertrophy along the TFL, gluteus medius, and gluteus minimus muscles. The mass appeared more hyperechoic than adipose tissue, with its thickest point measuring approximately 4 cm. This finding prompted further evaluation. 

**Figure 1:**
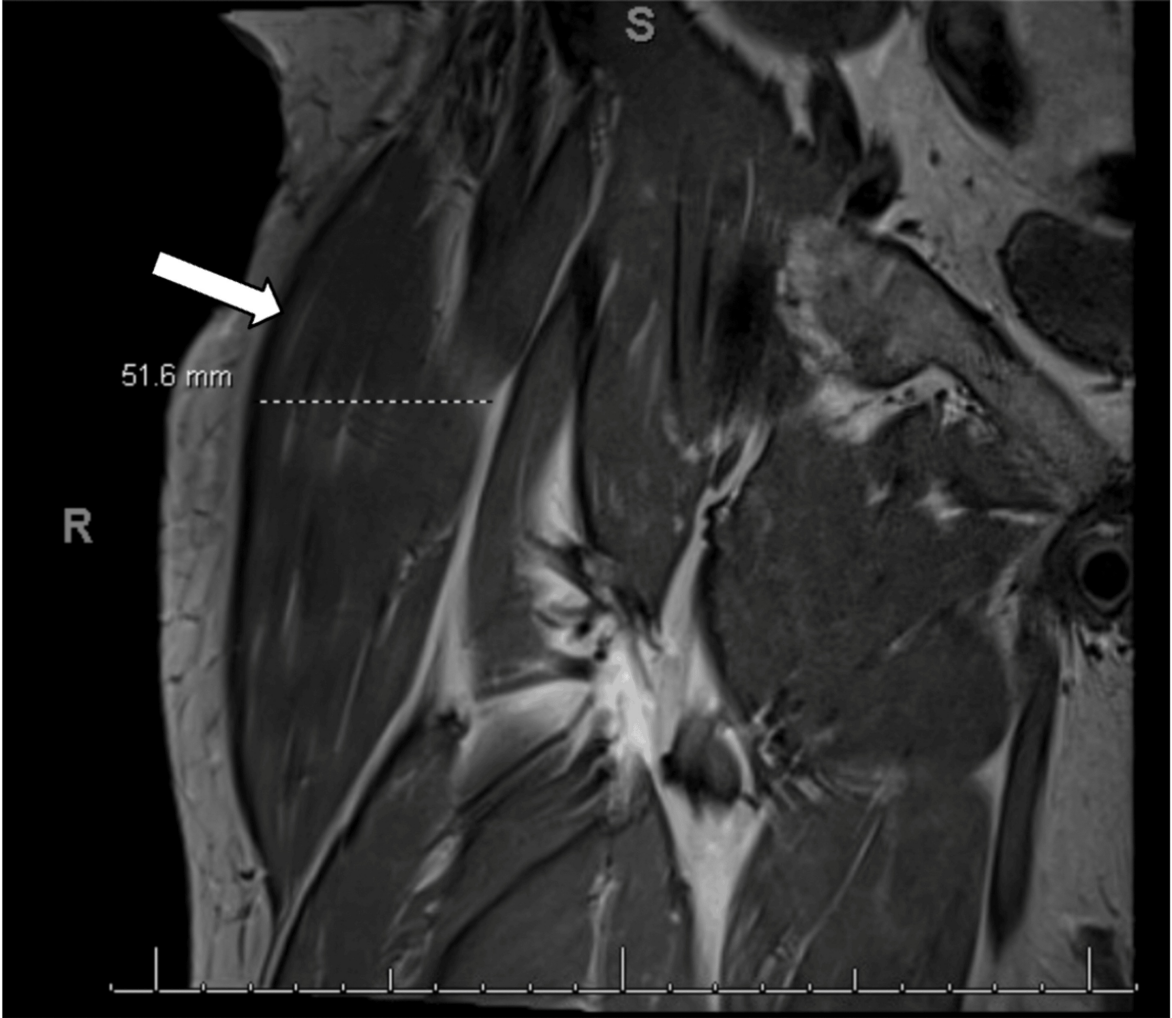
MRI showing hypertrophy of the TFL The scan of the right lower extremity in the coronal plane shows an enlarged TFL muscle measuring 51.6 mm as demarcated in the image (white arrow). TFL, tensor fascia lata; MRI, magnetic resonance imaging

**Figure 2:**
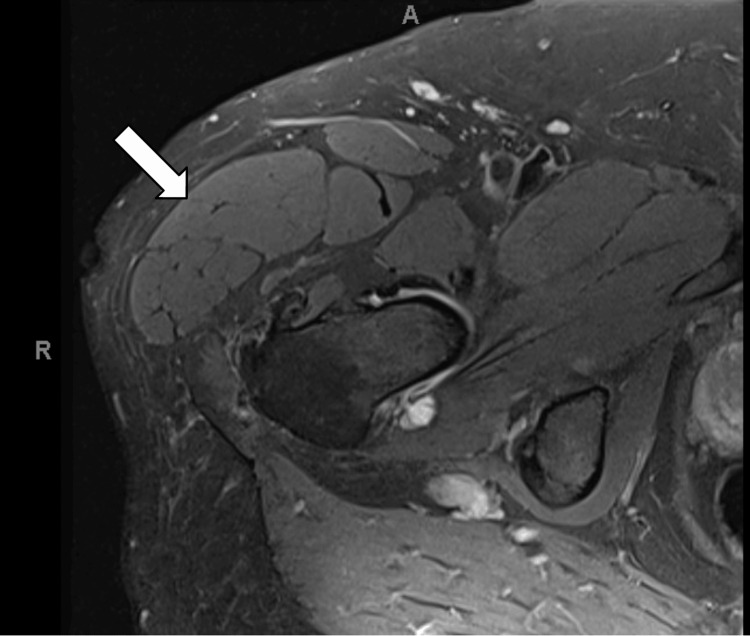
MRI showing a cross-section of TFL hypertrophy The scan of the right lower extremity at the level of the hip in the axial plane shows the enlargement of the TFL muscle (white arrow). TFL, tensor fascia lata; MRI, magnetic resonance imaging

Subsequent MRI of the hip revealed a large TFL with a diameter of up to 5.2 cm, as seen in Figure 1 and Figure 2. The hypertrophied TFL demonstrated homogeneous muscle signal intensity on all sequences without evidence of a discrete mass, abnormal enhancement, necrosis, or infiltrative features, making a soft tissue tumor unlikely. While the etiology of TFL hypertrophy remains uncertain, we hypothesize a potential association with lumbar spinal stenosis radiculopathy based on limited reports in the literature, though the hip MRI alone does not establish this causal relationship. Diagnosed with pseudotumor syndrome of the TFL, the patient underwent ultrasound-guided TFL chemodenervation with 50 units of onabotulinum toxin type A, which significantly improved his symptoms. Follow-up visits confirmed a marked reduction in TFL muscle mass on physical examination along with improved pain; however, the patient continued to experience some residual pain and a sensation of fullness in the right leg. His condition was managed with increased doses of botulinum toxin injections, up to 100 units. At subsequent visits, the patient reported further improvement in pain over the TFL. Given the successful diagnosis and management of the patient's condition and symptoms, we plan to closely monitor the patient for potential further intervention for the pseudotumor syndrome of the TFL and ongoing management of lumbar stenosis.

## Discussion

We present a case of a 65-year-old male with lower back and hip pain in the setting of lumbar canal and foraminal stenosis, and likely mechanical gluteal dysfunction, who developed pain, swelling, and fullness of the right lateral gluteal region. These symptoms were caused by hypertrophy of the TFL. This case is significant as it adds to the limited body of existing literature describing TFL hypertrophy and its possible etiologies. There is substantial overlap in the clinical presentation of TFL hypertrophy and the more serious diagnosis of sarcoma, with both conditions presenting as an anterolateral thigh mass. Therefore, increasing clinical awareness of the potential diagnosis of TFL hypertrophy or pseudohypertrophy in relevant cases is important. 

The etiology of this condition is not entirely clear, but two possible mechanisms suggested in the literature are mechanical and denervation hypertrophy [1]. Mechanically, hypertrophy can follow local muscle tears, hip osteoarthritis, or anterior cruciate ligament tears [3]. Hypertrophy can also present in the setting of gluteus medius and minimus muscle atrophy, potentially as a compensatory mechanism for hip abduction function [3]. Additionally, altered gait and biomechanics following spine or hip surgery can cause the condition. Denervation hypertrophy, on the other hand, occurs when a muscle paradoxically enlarges, rather than atrophies, in response to loss of innervation [2]. This pathology can occur in various scenarios including radiculopathies, peripheral nerve injuries, peripheral neuropathies, and disorders of the anterior horn cells, such as poliomyelitis [4]. In rare instances, TFL pseudohypertrophy has been reported, characterized by fatty and connective tissue infiltration into the muscle belly, rather than true hypertrophy, which involves an increase in muscle fiber volume [5]. The exact mechanisms leading to pseudohypertrophy are not completely understood, and some cases are idiopathic, suggesting the process may be independent of the mechanisms causing true hypertrophy [5]. In our case, TFL hypertrophy was present in the setting of both denervation and mechanical hypertrophy. The denervation was likely related to the patient’s L4 radiculopathy, confirmed by MRI findings of moderately severe foraminal narrowing at the L4-L5 level. The mechanical contribution likely involved altered gait mechanics secondary to back pain, radiculopathy, and gluteal dysfunction.

Limited case reports and series in the literature discuss TFL hypertrophy or pseudohypertrophy, and many describe patients with clinical symptoms or imaging findings similar to those in our case. One existing case written by Shields et al. describes similar US and MRI findings of unilateral TFL muscle hypertrophy to those found in our case [1]. For both Shields's case, as well as another case described by Pereira et al., sarcoma is the suspected diagnosis until MRI imaging points clinicians in the direction of TFL hypertrophy, rather than malignancy, due to muscle enlargement without fatty replacement, edema, or abnormal enhancing masses [1,3]. For TFL hypertrophy, MRI is the gold standard for confirming the diagnosis in nearly all cases [1,3,5-9,10,11]. In our case, we used both diagnostic ultrasound and MRI as diagnostic tools. However, ultrasound as a modality can be highly user-dependent, making MRI the preferred gold standard. 

Treatment for TFL hypertrophy often involves physical therapy consisting of stretching and strengthening of the hip abductors, although patients may also benefit from botulinum toxin injections [1]. Strengthening of the hip abductors, namely the gluteus medius and minimus muscles, may help to address the compensatory mechanical causes of TFL hypertrophy in response to weakened overall hip abduction function. In our case, the patient was treated with increasing doses of botulinum toxin, which resulted in a reduction of muscle mass and an improvement in pain symptoms.

## Conclusions

A pseudotumor of the TFL is a rare condition characterized by hypertrophy of the TFL, typically presenting as a mass in the anterolateral thigh and accompanied by localized pain. This condition can mimic the presentation of a soft tissue tumor. Clinical assessment and MRI characteristics are crucial for an accurate diagnosis. To limit unnecessary diagnostic testing and invasive procedures, clinicians should be aware of TFL hypertrophy and its possible etiologies.
